# Serologic control against hepatitis B virus among dental students of the University of Granada, Spain

**DOI:** 10.4317/medoral.20579

**Published:** 2015-08-04

**Authors:** María Teresa Arias-Moliz, Laura Rojas, Francisco Liébana-Cabanillas, Carmen Bernal, Francisca Castillo, Alberto Rodríguez-Archilla, Ana Castillo, José Liébana

**Affiliations:** 1 DDS, PhD. Assistant Professor. MD, BDS. Microbiologist. MD, PhD. Associate Professor. BNS. Technical assistant. MD, PhD. Associate Professor. MD, PhD. Professor. Department of Microbiology, Schools of Dentistry and Medicine. University of Granada, Spain; 2 PhD. Assistant Professor. Department of Marketing and Market Research, School of Economics and Business Administration. University of Granada. Spain; 3 MD, PhD. Associate Professor. Department of Stomatology, School of Dentistry. University of Granada, Spain

## Abstract

**Background:**

To evaluate the immunological situation against hepatitis B virus (HBV) of a cohort of dentistry students, to analyze the behavior of the levels of hepatitis B surface antigen (anti-HBs) after the administration of one or three vaccine doses, and to determine the influence of age and sex on the immune response.

**Material and Methods:**

This retrospective cohort study included students attending the School of Dentistry of the institution where the study was performed from 2005 to 2012 who had completed the public health vaccination calendar for HBV at the age of 12-13. Data on age, sex, basal anti-HBs levels, post-vaccination anti-HBs results and final anti-HBs levels were collected. Comparisons of the basal and final levels, as well as associations regarding age and sex, were performed by means of the Student t and Chi-square tests.

**Results:**

Of the 359 students, 97 (27.02%) had basal antibody concentrations <10 mIU/ml, whereas in 262 the levels of anti-HBs were ≥10 mIU/ml (72.98%). Of the 288 participating students who completed the School´s protocol for immunization, 287 (99.65%) attained a level of protection ≥10 mIU/ml. Globally, there were statistically significant differences between the basal antibody levels and those achieved after administration of the vaccine and booster, but no association with age or sex was observed.

**Conclusions:**

About 70% of dental students vaccinated as preadolescents had serologic evidence of protection against HBV. Administering a booster is associated with the presence of an excellent immune memory. There is clearly a need to reinforce control of the antibody levels in groups at risk, such as Dentistry students.

**Key words:**Dental students, hepatitis B virus, serologic control.

## Introduction

Infection with the hepatitis B virus (HBV) is a public health problem in terms of morbidity and mortality. It is estimated that two billion people worldwide have been infected with HBV and about 600,000 people die every year due to the consequences of hepatitis B, mostly from cirrhosis and hepatocellular carcinoma (1).

Strategies for controlling the incidence of HBV infection combine preventive measures and universal vaccination among newborns and adolescents. After primary immunization with the hepatitis B vaccine, the titer of antibody to hepatitis B surface antigen (anti-HBs) considered seroprotective is ≥10 milli-international units per milliliter (mIU/ml) (2). The levels of anti-HBs decline over time, however, and many people previously vaccinated may have anti-HBs below the accepted threshold of protection when tested 10-15 years after the primary series (3,4). At present there is controversy regarding the need to administer an additional vaccine dose (booster) in immunocompetent individuals previously vaccinated yet whose levels of anti-HBs have decreased due to the existence of immune memory (4).

Dentists comprise a high risk group in the face of HBV infection, given their frequent exposure to blood or body fluids containing HBV. This risk is higher during the professional training period (5). Since the introduction of obligatory vaccination against HBV for healthcare personnel in 1991, the occupational risk in dentists has been minimized (6,7). Notwithstanding, some individuals are not correctly vaccinated, and a low proportion of cases may show failure in the immune response (8). Moreover, in Spain, post-vaccination serological testing is not carried out; hence it is impossible to determine the level of initial response to the vaccine, which appears to be the main predictor of anti-HBs persistence (9-11). In view of this background, the objective of this retrospective study was to evaluate the immunological situation against HBV of a cohort of students at the School of Dentistry of the institution where the study was performed, to analyze the behavior of anti-HBs levels after the administration of a full dose of vaccine or a booster, and to determine the influence of age and sex on immune response.

## Material and Methods

This retrospective cohort study was carried out at the School of Dentistry. Eligible people were students attending from 2005 to 2012 who had completed the public health vaccination calendar for HBV at the age of 12-13. Data on students regarding age and the sex were collected, as well as basal anti-HBs levels, post-vaccination anti-HBs results and final anti-HBs levels. The Ethics Committee of Human Research of the institution where the study was performed approved the protocol. All subjects signed the informed consent form.

- School of Dentistry Hepatitis B immunization protocol

Basal anti-HBs titers were determined through chemioluminiscence (Anti-HBs Architect System, Abbott, Chicago, USA) when the students were attending their second undergraduate year in the Dentistry program. According to the basal levels, the students were designated as non-immune (anti-HBs <10 mIU/ml) and immune (anti-HBs ≥10 mIU/ml). On the basis of their level of protection, the immune students were, in turn, divided into low (anti-HBs 10-99 mIU/ml), medium (anti-HBs 100-1000 mIU/ml) or high level of protection (anti-HBs >1000 mIU/ml). In view of the basal levels of anti-HBs, the students with antibodies <10 mUI/ml received a full vaccine series with three doses of Engerix-B 20 µg/ml (GlaxoSmithKline Biologicals, Rixensart, Belgium) according to a schedule of 0, 1 and 6 months. When the levels were 10-99 mIU/ml, just one additional vaccine dose was administered. In either case, post-vaccination testing was performed three months after administration of the last dose. If a student´s level of immunization remained low (anti-HBs 10-99 mIU/ml) after vaccination with either one or three vaccine doses, an additional booster dose was repeated. Post-vaccination testing was performed three months after administration of the booster. Those subjects with basal levels of ≥100 mIU/ml were monitored at 2-3 years’ time.

- Statistical analysis

All statistical analyses were performed by means of SPSS 15.0 software (SPSS Inc., Chicago, IL, USA). Descriptive techniques were used for the sample and the comparison of means. The Chi-square test was used to evaluate the significance of differences in anti-HBs levels with regard to age and sex. The Student t-test was used to compare the anti-HBs basal and final levels. The level of statistical significance was set at *P*<0.05. The presence of immune memory was defined as an anti-HBs titer ≥4fold greater post- than pre-booster.

## Results

The data analyzed were from 359 students attending the School of Dentistry during 2005-2012 and who had been vaccinated at the age of 10-12 years old. Of the 359 students, 97 (27.02%) had basal antibody concentrations <10 mIU/ml. In 262 the levels of anti-HBs were ≥10 mIU/ml (72.98%); the concentration was 10-99 mIU/ml in 129 of these students (35.93%), and it was >100 mIU/ml in the remaining 133 (37.05%). The mean age of students was 20.09 years, with a standard deviation of 3.26. In 94.4% the age was less than or equal to 23, and the most frequent age was 19 (72.1%). By sex, 262 were female (72.9%) and 97 were male (27.01%). The differences observed between anti-HBs basal levels and the age and sex of participants were not statistically significant.

**Figure 1:**
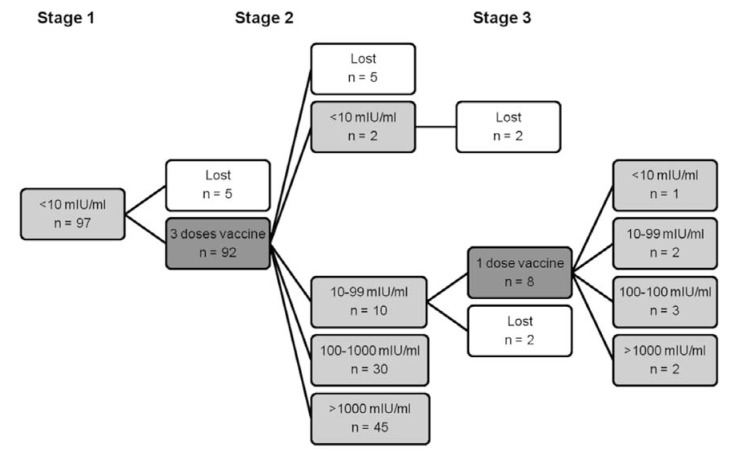
Students with basal levels of anti-HBs <10 mIU/ml. Number of students (n) and anti-HBs levels in the different phases of the School of Dentistry Hepatitis B immunization procedure. Stage 1: basal anti-HBs levels. Stage 2: vaccination with 3-dose vaccine and serologic test 3 months after the last dose. Stage 3: vaccination with one additional vaccine dose when anti-HBs levels were 10-99 mIU/ml, and serologic test three months after the last dose.

Of the 97 non-immune students (Fig. 1), 92 were revaccinated and 87 underwent the post-vaccination testing; levels of anti-HBs ≥10 mIU/ml were obtained in 85 students (97.70%). The two subjects who maintained anti-HBs levels <10 mIU/ml did not continue the procedure, meaning a fourth dose was not administered to them. After administration of a booster to those subjects who had had anti-HBs levels in the range 10-99 mIU/ml after receiving the vaccine, in one case the levels dropped to <10 mIU/ml, in two cases the levels remained in the same range, and in the rest the levels went up to ≥100 mIU/ml. In this group, 14 subjects did not complete the protocol.

**Figure 2:**
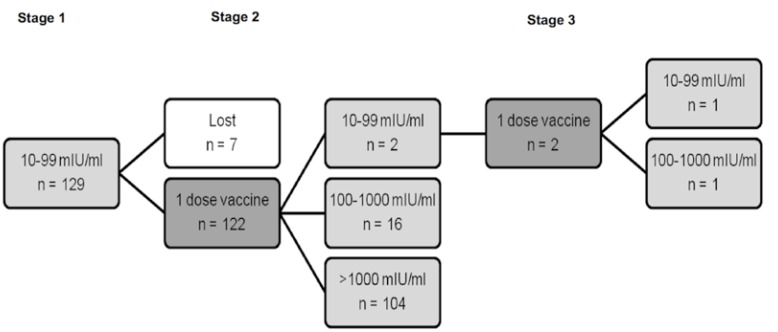
Students with basal levels of anti-HBs 10-99 mIU/ml. Number of students (n) and anti-HBs levels in the different phases of the School of Dentistry Hepatitis B immunization procedure. Stage 1: basal anti-HBs levels. Stage 2: vaccination with 1-dose vaccine and serologic test three months afterwards. Stage 3: vaccination with one additional vaccine dose when anti-HBs levels were 10-99 mIU/ml and serologic test three months after the last dose.

Meanwhile, in 129 students the basal levels of anti-HBs were 10-99 mIU/ml (Fig. 2). Seven of these students chose to discontinue the protocol and 122 were given one or two boosters, leading to antibody concentrations of ≥100 mIU/ml in 121 students (99.18%).

**Figure 3:**
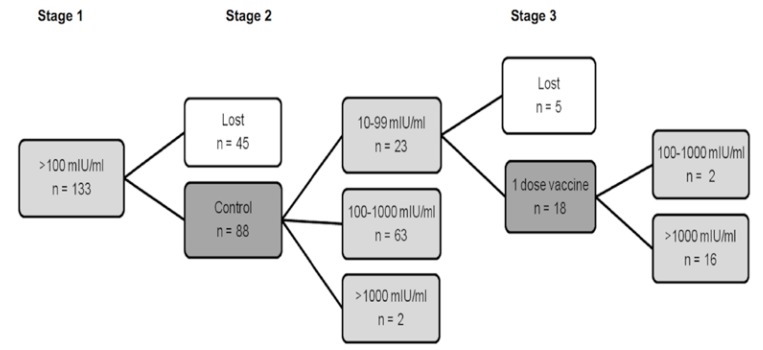
Students with basal levels of anti-HBs ≥100 mIU/ml. Number of students (n) and anti-HBs levels in the different phases of the School of Dentistry Hepatitis B immunization procedure. Stage 1: basal anti-HBs levels. Stage 2: serologic test 2-3 years afterwards. Stage 3: vaccination with one additional vaccine dose when anti-HBs levels were 10-99 mIU/ml, and serologic test three months after the last dose.

Finally, out of the 133 students who initially had a basal anti-HBs level of ≥100 mIU/ml, 50 did not complete the immunization protocol, and of those who finished it, 65 (78.31%) did so with values between 100 and 1000 mIU/ml, while the remaining 18 (21.69%) gave levels of >1000 mIU/ml (Fig. 3).

In short, during the study 71 students (19.78%) abandoned the procedure in one of its stages. Of the 288 subjects completing the immunization protocol, 287 (99.65%) achieved levels of protection of ≥10 mIU/ml: three of them (1.04%) attained a low level of protection of 10-99 mIU/ml, 115 (39.93%) achieved intermediate levels of 100-1000 mIU/ml, and 169 students (58.68%) had high protective levels, over 1000 mIU/ml. Globally, there were statistically significant differences between the basal antibody levels and those obtained after administration of the vaccine and booster. The presence of immune memory was confirmed in the 145 students (96.67%) of a total of 150 that received a booster dose, as an increase in antibody levels of 4 times or more was observed.

There were no significant differences in the pattern of response after the revaccination or the booster dose when the findings were distributed by sex and age groups (*P*>0.05).

## Discussion

The prevalence of HBV markers increases over time in conjunction with the dental practice of professionals, which confirms that there is contact with the virus, and therefore a chance of infection (12). For this reason, it is important that dentists be considered at risk and be protected during their entire professional careers. The immunization protocol at the School of Dentistry of the institution aims to monitor and ensure protection against HBV before the students actually engage in clinical practices at the School (13) or go out into the working world (5,14,15). Data were gathered from a total of 359 undergraduates who had received the HBV vaccine as pre-adolescents, but had not undergone post-vaccination serologic testing. Such testing is recommended and proves cost-effective among healthcare personnel at a risk of infection by HBV, since they may often need prophylactic measures after accidental percutaneous and/or cutaneomucosa exposure (16).

Approximately seven years after their primary vaccination, 72.98% of the students still had detectable antibodies above the seroprotective titer, whereas in the other 27.02% the basal level of anti-HBs was below this threshold. The decrease in antibody levels may be explained by an initial lack of response to the vaccine in these particular individuals for such reasons as smoking habit, obesity, genetic factors, or immune suppression (17-20), or else because they responded to the primary 3-dose vaccination series but the anti-HBs gradually declined over time. It is known that an estimated 13%-60% of initial responders to the HBV vaccine may lose detectable anti-HB in subsequent years (3,5).

Much debate surrounds the number of doses of vaccine that should be administered when the levels of anti-HBs after vaccination are <10 mIU/ml. Some authors affirm that a single dose is sufficient to reach the level of seroprotection (4), while others insist on the need to apply a full dose of vaccine (21,22). Following the recommendations by the Centers for Disease Control and Prevention for healthcare personnel (5), and because the initial response to the vaccine is not known, the School of Dentistry protocol specifies revaccination of the non-immune students with a triple dose. The level of seroprotection obtained in this group was 97.70% (85/87). These results are consistent with previous studies and evidence the immediate efficacy of the vaccine (15,21,22).

The immune students whose levels of anti-HBs were low, that is 10-99 mIU/ml, received a dose of vaccine in order to attain levels over 100 mIU/ml. The administration of a booster dose of vaccine showed a rapid increase, fourfold or greater, of the anti-HBs concentration in 145 of the 150 students in this group. The response to the booster dose indicates the presence of an HBV-specific immune memory after reception of a vaccine, which is known as immune response (11,23). These results confirm findings from previous studies, and come to underline that there would be no need to administer a booster to individuals with low levels of anti-HBs within an interval of approximately seven years (4,24). However, several studies report cases of failure of the immune memory 15 years after reception of the primary vaccination series, implying that the duration of immune memory is not well established (7,25). Such findings urge us to reflect upon the need for periodical serologic monitoring of certain groups at risk, and the risk groups would clearly include healthcare students (26).

The 26.13% of subjects whose anti-HBs basal levels were ≥100 mIU/ml showed a decrease in antibodies 2-3 years later. This finding supports the decay of antibodies over time since vaccination (1,27,28) and once again points to the importance of controlling anti-HBs levels, especially in high-risk groups. Globally, we could not establish any gender or age differences in the basal levels of anti-HBs or in the immunological response to HBV vaccine and booster; other authors arrive at similar conclusions (24,29,30). The fact that 72.1% of the students were 19 years of age would justify the lack of significant differences when comparing the anti-HBs levels and age. However, if we compare the final levels of anti-HBs and sex, women show a somewhat higher level of protection than men.

The drop out rate of the serological immunization protocol was found to be greater among the subjects with basal anti-HBs titers ≥100 mIU/ml, which is logical in view of their high level of protection against HBV infection. Of the 288 students who completed the immunization procedure, 287 (99.65%) exhibited a level of antibodies over the seroprotective threshold, and 284 of these (98.61%) had a concentration of ≥100 mIU/ml. Noteworthy is the case of one non-immune student who, after administration of a 3-dose vaccine followed by a booster, gave anti-HBs levels under the seroprotective threshold. The absence of HBsAg was confirmed. This poor immune response could have to do with immunosuppression or with genetic factors (16,18,19).

A limitation of this study that must be addressed is that data on initial vaccination against HBV was collected by self-report, and was not veriﬁed by medical records; this introduces a possibility of bias in the basal antibody levels registered.

## Conclusions

Within the limitations of this study, our findings indicate that about 70% of students vaccinated as pre-adolescents showed serologic evidence of protection against HBV after approximately seven years. Moreover, almost all non-immune students were seroprotected after revaccination. Administering a booster is associated with the presence of an excellent immune memory in most vaccinees. Thus far we are not certain of the clinical relevance of immune memory, however. There is clearly a need to reinforce control of the antibody levels in groups at risk, such as Dentistry students.

## References

[1] World Health Organization (2009). Hepatitis B vaccines WHO position paper. Weekly Epidemiological Record (WER).

[2] Chaves SS, Fischer G, Groeger J, Patel PR, Thompson ND, Teshale EH (2012). Persistence of long-term immunity to hepatitis B among adolescents immunized at birth. Vaccine.

[3] Honorati MC, Palareti A, Dolzani P, Busachi CA, Rizzoli R, Facchini A (1999). A mathematical model predicting antihepatitis B virus surface antigen (HBs) decay after vaccination against hepatitis B. Clin Exp Immunol.

[4] Leuridan E, Van Damme P (2011). Hepatitis B and the need for a booster dose. Clin Infect Dis.

[5] Advisory Committee on Immunization Practices; Centers for Disease Control and Prevention CDC (2011). Immunization of health-care personnel: recommendations of the Advisory Committee on Immunization Practices (ACIP). MMWR Recomm Rep.

[6] Petti S, Messano GA, Polimeni A (2011). Dentists' awareness toward vaccine preventable diseases. Vaccine.

[7] Yang SG, Wang B, Chen P, Yu CB, Deng M, Yao J (2012). Effectiveness of HBV vaccination in infants and prediction of HBV prevalence trend under new vaccination plan: findings of a large-scale investigation. PLoS One.

[8] Boot HJ, van der Waaij LA, Schirm J, Kallenberg CG, van Steenbergen J, Wolters B (2009). Acute hepatitis B in a healthcare worker: a case report of genuine vaccination failure. J Hepatol.

[9] Fitzsimons D, François G, Hall A, McMahon B, Meheus A, Zanetti A (2005). Long-term efficacy of hepatitis B vaccine, booster policy and impact of hepatitis B virus mutants. Vaccine.

[10] Duval B, Gîlca V, Boulianne N, De Wals P, Massé R, Trudeau G (2005). Comparative long term immunogenicity of two recombinant hepatitis B vaccines and the effect of a booster dose given after five years in a low endemicity country. Pediatr Infect Dis J.

[11] Gilca V, De Serres G, Boulianne N, Murphy D, De Wals P, Ouakki M (2013). Antibody persistence and the effect of a booster dose given 5, 10 or 15 years after vaccinating preadolescents with a recombinant hepatitis B vaccine. Vaccine.

[12] Zanetti AR, Tanzi E, Pozzi A, Romano L, Bergamini F (1990). Yeast-derived hepatitis B vaccine in dental students. A three-year follow-up study. Vaccine.

[13] Centers for Disease Control and Prevention CDC (1997). Immunization of health-care workers: recommendations of the Advisory Committee on Immunization Practices (ACIP) and the Hospital Infection Control Practices Advisory Committee (HICPAC). Morbidity and Mortality Weekly Report.

[14] Lindley MC, Lorick SA, Spinner JR, Krull AR, Mootrey GT, Ahmed F (2011). Student vaccination requirements of U.S. health professional schools: a survey. Ann Intern Med.

[15] Spradling PR, Williams RE, Xing J, Soyemi K, Towers J (2012). Serologic testing for protection against hepatitis B virus infection among students at a health sciences university in the United States. Infect Control Hosp Epidemiol.

[16] van Wijk PT, Meiberg AE, Bruers JJ, Groenewold MH, van Raalten AL, Dam BA (2012). The risk of blood exposure incidents in dental practices in the Netherlands. Community Dent Oral Epidemiol.

[17] Averhoff F, Mahoney F, Coleman P, Schatz G, Hurwitz E, Margolis H (1998). Immunogenicity of hepatitis B Vaccines. Implications for persons at occupational risk of hepatitis B virus infection. Am J Prev Med.

[18] Shaw FE, Guess HA, Roets JM, Mohr FE, Coleman PJ, Mandel EJ (1989). Effect of anatomic injection site, age and smoking on the immune response to hepatitis B vaccination. Vaccine.

[19] Weber DJ, Rutala WA, Samsa GP, Santimaw JE, Lemon SM (1985). Obesity as a predictor of poor antibody response to hepatitis B plasma vaccine. JAMA.

[20] Hennig BJ, Fielding K, Broxholme J, Diatta M, Mendy M, Moore C (2008). Host genetic factors and vaccine-induced immunity to hepatitis B virus infection. PLoS One.

[21] Alper CA, Kruskall MS, Marcus-Bagley D, Craven DE, Katz AJ, Brink SJ (1989). Genetic prediction of nonresponse to hepatitis B vaccine. N Engl J Med.

[22] Craven DE, Awdeh ZL, Kunches LM, Yunis EJ, Dienstag JL, Werner BG (1986). Nonresponsiveness to hepatitis B vaccine in health care workers. Results of revaccination and genetic typings. Ann Intern Med.

[23] Are booster immunisations needed for lifelong hepatitis B immunity (2000). European Consensus Group on Hepatitis B Immunity. Lancet.

[24] Aypak C, Yüce A, Yıkılkan H, Görpelioğlu S (2012). Persistence of protection of hepatitis B vaccine and response to booster immunization in 2- to 12-year-old children. Eur J Pediatr.

[25] Petersen KM, Bulkow LR, McMahon BJ, Zanis C, Getty M, Peters H (2004). Duration of hepatitis B immunity in low risk children receiving hepatitis B vaccinations from birth. Pediatr Infect Dis J.

[26] Tohme RA, Ribner B, Huey MJ, Spradling PR (2011). Hepatitis B vaccination coverage and documented seroprotection among matriculating healthcare students at an academic institution in the United States. Infect Control Hosp Epidemiol.

[27] Gilca V, De Serres G, Boulianne N, De Wals P, Murphy D, Trudeau G (2010). Antibody and immune memory persistence after vaccination of preadolescents with low doses of recombinant hepatitis B vaccine. Hum Vaccin.

[28] Poovorawan Y, Chongsrisawat V, Theamboonlers A, Bock HL, Leyssen M, Jacquet JM (2010). Persistence of antibodies and immune memory to hepatitis B vaccine 20 years after infant vaccination in Thailand. Vaccine.

[29] Tele SA, Martins RM, Lopes CL, dos Santos MA, Souza KP, Yoshida CF (2001). Immunogenicity of a recombinant hepatitis B vaccine (Euvax-B) in haemodialysis patients and staff. Eur J Epidemiol.

[30] Hammitt LL, Hennessy TW, Fiore AE, Zanis C, Hummel KB, Dunaway E (2007). Hepatitis B immunity in children vaccinated with recombinant hepatitis B vaccine beginning at birth: a follow-up study at 15 years. Vaccine.

